# Current Landscape and Emerging Fields of PET Imaging in Patients with Brain Tumors

**DOI:** 10.3390/molecules25061471

**Published:** 2020-03-24

**Authors:** Jan-Michael Werner, Philipp Lohmann, Gereon R. Fink, Karl-Josef Langen, Norbert Galldiks

**Affiliations:** 1 Department of Neurology, Faculty of Medicine and University Hospital Cologne, University of Cologne, Kerpener St. 62, 50937 Cologne, Germany; jan-michael.werner@uk-koeln.de (J.-M.W.); gereon.fink@uk-koeln.de (G.R.F.); 2 Institute of Neuroscience and Medicine (INM-3, -4), Research Center Juelich, Leo-Brandt-St., 52425 Juelich, Germany; p.lohmann@fz-juelich.de (P.L.); k.j.langen@fz-juelich.de (K.-J.L.); 3 Department of Nuclear Medicine, University Hospital Aachen, 52074 Aachen, Germany

**Keywords:** amino acid, FET, FACBC, FDOPA, immunoPET, molecular imaging, glioma, brain metastases

## Abstract

The number of positron-emission tomography (PET) tracers used to evaluate patients with brain tumors has increased substantially over the last years. For the management of patients with brain tumors, the most important indications are the delineation of tumor extent (e.g., for planning of resection or radiotherapy), the assessment of treatment response to systemic treatment options such as alkylating chemotherapy, and the differentiation of treatment-related changes (e.g., pseudoprogression or radiation necrosis) from tumor progression. Furthermore, newer PET imaging approaches aim to address the need for noninvasive assessment of tumoral immune cell infiltration and response to immunotherapies (e.g., T-cell imaging). This review summarizes the clinical value of the landscape of tracers that have been used in recent years for the above-mentioned indications and also provides an overview of promising newer tracers for this group of patients.

## 1. Introduction

For the management of patients with brain tumors, clinicians frequently need to rely on imaging information obtained from anatomical magnetic resonance imaging (MRI) before, during, and after the treatment. While contrast-enhanced MRI is of paramount value in neuro-oncology, its specificity for neoplastic tissue is low, and changes of the blood-brain barrier permeability as indicated by contrast enhancement are not limited to tumor tissue [1,2,3,4,5,6,7]. Nevertheless, precise delineation of tumor extent, including non-enhancing tumor subregions, is decisive for several diagnostic and therapeutic steps (e.g., planning of biopsy, surgery, or radiotherapy) [7,8]. Following radio- and/or chemotherapy, neurooncologists often encounter treatment-related changes. Some of these, e.g., pseudoprogression, are difficult to differentiate from actual tumor progression with conventional MRI alone [6,7,8,9,10,11,12]. Pseudoprogression describes a phenomenon characterized by an increase of contrast enhancement without clinical deterioration, and which disappears again over time without any treatment change [3,9,13,14,15,16]. Such treatment-related changes may occur early (in the case of pseudoprogression typically within the first 12 weeks after chemoradiation completion) or late (several months or even years after radiotherapy in the case 31 of radiation necrosis) [7,8,17].

Furthermore, surrogates of treatment response or progression obtained from MRI (e.g., a decrease of contrast enhancement or the fluid-attenuated inversion recovery (FLAIR) signal hyperintensity) may be unspecific. They can be influenced by inflammation, infarction, and reactive changes after surgery [2,6,12,18]. If treatment-related changes remain unidentified, an effective treatment may be erroneously terminated prematurely. The latter may also harm survival and mislead study results evaluating novel treatment approaches for tumor relapse [19].

To overcome these diagnostic challenges, imaging techniques with higher diagnostic accuracy than conventional MRI offering more than just anatomical information are needed. Apart from advanced MRI techniques, positron-emission tomography (PET) imaging has been evaluated over the past decades. It has been shown that PET imaging offers additional value in neuro-oncology since it enables the non-invasive evaluation of molecular and metabolic features of brain tumors. PET, therefore, is of great value for the indications mentioned above, which are of particular clinical interest [7,8,10,20]. Consequently, the PET task force of the Response Assessment in Neuro-Oncology (RANO) working group highlighted the additional clinical value of PET imaging using amino acid tracers compared to anatomical MRI. Accordingly, its widespread clinical use was recommended in patients with glioma and brain metastases [17,21].

The continuously growing landscape of PET tracers enables the evaluation of many biochemical processes in patients with brain tumors. With the advent of newer treatment options in neuro-oncology, in particular, targeted therapy and various immunotherapy options, the needs for additional information derived from neuroimaging in terms of characterization of the tumor environment, the evaluation of tumoral drug accumulation, immune cell infiltration, and the diagnosis of treatment-related changes following these newer treatment options are steadily increasing. Some of these requirements may be met by the existing landscape of well-established PET tracers, while others can be addressed by newer ones [22,23].

This review summarizes the value of PET tracers that have been used in brain tumors in recent years for the most relevant clinical indications. Furthermore, more unique but promising PET tracers are summarized and discussed.

## 2. Methods

A PubMed search using the terms “PET”, “positron”, “tracer”, “glioma”, “brain metastases”, “FDG”, “amino acid”, “methionine”, “FET”, “FDOPA”, “FACBC”, “AMT”, “TSPO”, “GE-180”, “FLT”, “FAZA”, “EGFR”, “VEGF”, “immunoPET”, “isocitrate dehydrogenase”, “radiotherapy”, “T-cell imaging”, “reporter gene”, “radiation necrosis”, “pseudoprogression”, “tumor extent”, “response assessment”, “treatment-related changes”, and combinations thereof was performed until January 2020. The PET tracers were evaluated regarding their clinical value for the delineation of tumor extent, diagnosis of treatment-related changes, the assessment of treatment response (Table 1), and according to the information provided by newer PET probes (Table 2). 

## 3. Current Landscape of PET Imaging

PET allows targeting metabolic and molecular processes in patients with brain tumors relevant to diagnosis, treatment, and prognosis that cannot be assessed with anatomic computed tomography (CT) or MR imaging. A variety of PET tracers have been evaluated predominantly in glioma patients or patients with brain metastases with the main focus on glucose metabolism, amino acid transport, proliferation, hypoxia, blood flow, or angiogenesis. This section will provide an overview of PET tracers for brain tumors that have been evaluated in human subjects in the last years, especially for those above mentioned highly relevant indications in clinical routine. An overview is presented in Table 1.

### 3.1. PET Imaging of Glucose Metabolism

[^18^F]-2-Fluoro-2-deoxy-D-glucose ([^18^F]FDG) is the most widespread PET tracer in nuclear medicine. In neoplastic tissue, the uptake of [^18^F]FDG reflects the increased expression of glucose transporters and hexokinase. The latter enzyme phosphorylates glucose and [^18^F]FDG. In the central nervous system, the physiologically high and varying uptake of [^18^F]FDG in healthy brain parenchyma hampers the accurate delineation of the brain tumor. This limits the diagnostic accuracy for the correct identification of treatment-related changes and assessment of treatment response in gliomas and brain metastases [17,21]. It has repeatedly been shown that the diagnostic accuracy of [^18^F]FDG regarding the differentiation of radiation-induced changes from glioma and brain metastases recurrence is inferior to other imaging modalities, including advanced MRI and amino acid PET [17,24,25,26]. However, [^18^F]FDG PET seems to be of value for the delineation of tumor extent and assessment of treatment response in patients with primary central nervous system (CNS) lymphoma [27,28,29,30].

### 3.2. PET Using Amino Acid PET Tracers

**Figure 1:**
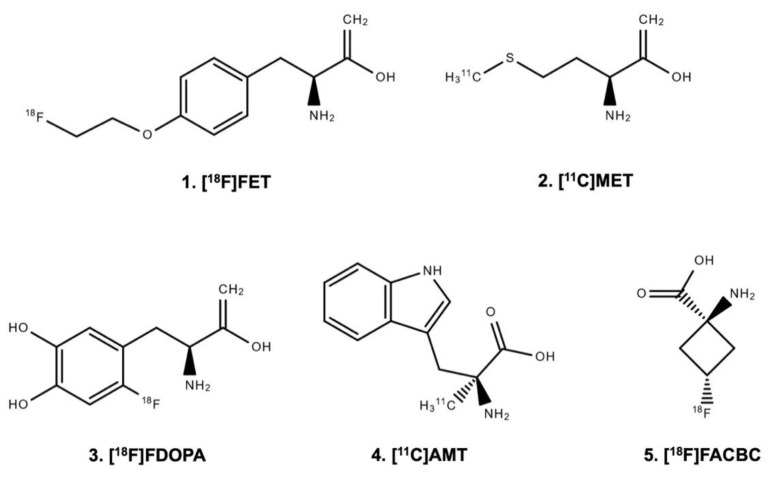
Chemical structure of radiolabeled amino acids.

Radiolabeled amino acid tracers (Figure 1) are of great interest in brain tumor imaging because of the high tumor-to-brain contrast based on the relatively high specificity for neoplastic tissue and the low uptake in healthy brain tissue [7,8,21,31,32,33].

#### 3.2.1. Uptake Mechanisms of Amino Acid PET Tracers

The uptake of the amino acid tracers O-(2-[^18^F]fluoroethyl)-L-tyrosine ([^18^F]FET), [^11^C]methyl-L-methionine ([^11^C]MET), and 3,4-dihydroxy-6-[^18^F]fluoro-L-phenylalanine ([^18^F]FDOPA) is mainly based on the increased expression of large neutral amino acid transporters of the l-type (LAT) in gliomas and brain metastases (i.e., subtypes LAT1 and LAT2) [7,34,35,36,37]. Moreover, LAT1 overexpression correlates with malignant phenotypes and proliferation of gliomas. It is associated with glioma angiogenesis [38,39]. A critical consideration for the practical application of [^11^C]MET compared to [^18^F]FET or [^18^F]FDOPA is the half-life of the [^11^C]-isotope ([^18^F] 110 vs. [^11^C] 20 min) [40,41], which allows the transport of [^18^F]FET and [^18^F]FDOPA to PET facilities. In contrast, the use of [^11^C]MET necessitates an on-site cyclotron. In many European centers, this logistical disadvantage has led to the replacement of [^11^C]MET predominantly by [^18^F]FET [7]. When using [^18^F]FDOPA, the physiological uptake in the striatum may hamper the evaluation of tumor extent [7,42].

The L-tryptophan analogue α-[^11^C]-methyl-L-tryptophan ([^11^C]AMT) is another radiolabeled amino acid with uptake via the LAT system. Additionally, [^11^C]AMT uptake is mediated via the kynurenine pathway and has a rate-limiting enzyme indoleamine 2,3-dioxygenase [43]. Indoleamine 2,3-dioxygenase is upregulated in various cancers including gliomas [44], which prompted the use of [^11^C]AMT PET in patients with brain tumors [45].

Other tracers such as the synthetic amino acid analog anti-1-amino-3-[^18^F]fluorocyclobutane-1-carboxylic acid ([^18^F]FACBC or [^18^F]fluciclovine) are also LAT-mediated but use additionally the alanine, serine, and cysteine transporter 2, which is upregulated in many human cancers [46,47,48,49].

#### 3.2.2. Value of Amino Acid PET Tracers for Brain Tumor Patients

For the planning of diagnostic and therapeutic procedures, the precise delineation of tumor spread is essential. For example, the tumor extent as assessed by amino acid PET provides valuable information for planning stereotactic biopsies, resection, and radiotherapy [1,50,51,52]. For [^11^C]MET and [^18^F]FET, it has been shown that the delineation of tumor extent, particularly in non-enhancing gliomas, can be assessed with high accuracy using amino acid PET [51,53]. Preliminary data suggest that newer tracers such as [^18^F]FACBC PET are also helpful in identifying metabolically active and non-contrast enhancing tumor regions in glioma patients [54,55,56]. Moreover, it has been shown that in the majority of cases, the metabolically active tumor burden as assessed by amino acid PET extends considerably beyond the volume of MRI contrast enhancement, which is of significant relevance for subsequent treatment planning [1,7,53,57]. Regarding the comparability of PET tracers for this indication, [^11^C]MET, [^18^F]FET, and [^18^F]FDOPA seem to be equally informative [58,59,60,61]. Nevertheless, it has to be pointed out that 20–30% of grade II gliomas, according to the World Health Organization (WHO) classification of tumors of the central nervous system [62,63], show no amino acid uptake [64,65,66]. A negative amino acid PET, therefore, does not exclude glioma [8].

**Figure 2:**
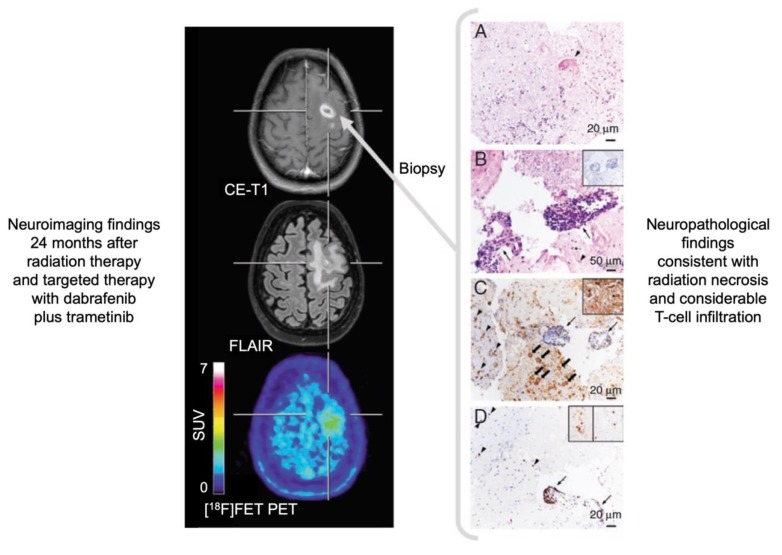
Radiation necrosis and chronic inflammation in a patient with brain metastases of a B-Raf proto-oncogene (BRAF)-mutated malignant melanoma who had been treated with whole-brain radiation therapy combined with concurrent dabrafenib plus trametinib. Twenty-four months later, the contrast-enhanced magnetic resonance imaging (MRI) indicates a recurrence of the brain metastases (left panel), whereas the O-(2-[^18^F]fluoroethyl)-L-tyrosine ([^18^F]FET) positron-emission tomography (PET) shows only insignificant metabolic activity and is consistent with the findings of treatment-related MRI changes. Neuropathological findings (right panel) after stereotactic biopsy show signs of radiation necrosis as well as considerable T-cell infiltration. (**A**) Hyaline, eosinophilic necrosis with evidence of a necrotic vessel wall (arrowhead). (**B**) Vital brain parenchyma besides necrosis with activated microglia cells (arrowhead), and blood vessels with lymphocyte infiltrates (arrows) without evidence of tumor cells (inserted box). (**C**) Adjacent to inflamed blood vessels (arrows), a resorption of necroses by macrophages (block arrows) as well as activated microglia cells (arrowheads) and astrocytes in the brain parenchyma (inserted box). (**D**) The main population of intra- and perivascular T-cell infiltrates are CD3^+^ (arrow), but also CD4^+^ (inserted box left) and CD8^+^ (inserted box right) T-cells contribute to the infiltrates (modified from Galldiks et al. [10], with permission from Oxford University Press).

For the differentiation of treatment-related changes from tumor relapse, amino acid PET also provides valuable diagnostic information. Using [^18^F]FET or [^18^F]FDOPA, especially the differentiation of radiation injury from tumor relapse in glioma patients, as well as in patients with brain metastases, can be obtained with a relatively high diagnostic accuracy between 80–90% (Figure 2) [11,26,67,68,69,70,71,72,73,74,75,76,77,78,79,80]. Importantly, in glioma patients, parameters derived from dynamic [^18^F]FET PET acquisition may further increase the diagnostic accuracy [67,74,75,76,78]. This has also been demonstrated in patients with brain metastases who underwent radiosurgery for brain metastases treatment [80,81]. The diagnostic accuracy of [^11^C]MET PET regarding this clinical question is slightly lower (approximately 75%) [8,82,83], which is most probably related to the higher affinity of [^11^C]MET for inflammation [84]. First PET studies using [^11^C]AMT or [^18^F]FACBC suggest that these tracers may also be of value for the differentiation of radiation injury from glioma progression [85,86].

The advent of immunotherapy using immune checkpoint inhibitors and targeted therapy has improved the survival of cancer patients, particularly in melanoma and lung cancer. Recent trials suggest that patients with brain metastases from these tumor entities may also benefit from these agents alone or in combination [87]. Regarding patients with brain metastases treated with checkpoint inhibitors or targeted therapy (frequently combined with radiotherapy), initial data indicate that amino acid PET may provide valuable information for differentiating relapse from equivocal MRI findings related to immunotherapy-induced inflammation [10,88].

Recently, a variety of experimental treatment options has been introduced for treating patients with high-grade glioma. [^18^F]FET PET was shown to differentiate benign MRI findings related to these experimental therapies, e.g., immunotherapy with dendritic cell vaccination or targeted therapy with regorafenib, from tumor relapse [89,90]. However, the number of patients treated with these therapies and monitored with [^18^F]FET PET is still small, and the results should be interpreted with caution.

The assessment of the response to a particular neurooncological treatment is of clinical relevance since treatment decisions can be negatively affected by treatment-related changes. The accurate assessment of response helps both to discontinue an ineffective treatment option as early as possible and to prevent an effective treatment from being erroneously terminated prematurely with a potentially harmful influence on survival. Furthermore, the evaluation of response also helps to avoid possible treatment side effects, e.g., bone marrow depression or fatigue, and, therefore, to maintain or even improve life-quality. It has been shown in glioma patients that the assessment of response to alkylating chemotherapy (i.e., temozolomide or lomustine) using [^11^C]MET or [^18^F]FET PET provides valuable additional information compared to contrast-enhanced MRI. Importantly, metabolic PET responders (i.e., patients with a decrease of tumor-to-brain ratios or metabolically active tumor volumes at follow-up relative to baseline imaging) had a significantly longer survival than metabolic non-responders [91,92,93,94,95,96,97,98].

Following antiangiogenic therapy using bevacizumab, the use of reduced contrast enhancement as a surrogate marker for treatment response is not optimal due to a phenomenon called pseudoresponse [9]. Pseudoresponse describes a decrease of contrast enhancement related to a rapid restoration of the blood-brain barrier by antiangiogenic drugs [9]. However, a clinical benefit is not infrequently lacking in patients with an impressive radiological response (pseudoresponse). [^18^F]FET and [^18^F]FDOPA PET may provide valuable information regarding the identification of pseudoresponse [99,100,101,102]. Moreover, [^18^F]FDOPA and [^18^F]FET PET were also able to predict a favorable clinical outcome in bevacizumab responders [101,102,103,104].

After chemoradiation completion, newly diagnosed glioblastoma patients can be treated with tumor-treating fields therapy in addition to adjuvant temozolomide chemotherapy [105]. Initial studies suggest that amino acid PET might identify responding patients undergoing tumor-treating fields therapy, but it has to be considered that the response can also be related to the concurrently applied chemotherapy or delayed chemoradiation effects [106,107].

### 3.3. PET Imaging of the Mitochondrial Translocator Protein

PET ligands targeting the 18 kDa mitochondrial translocator protein (TSPO), located at the outer mitochondrial membrane and formerly known as the peripheral benzodiazepine receptor, are also of interest in neuro-oncology [108,109]. TSPO is associated with neuroinflammation due to its expression in activated microglia, endothelial cells, and infiltrating macrophages [109]. The PET ligand [^11^C]PK11195 was one of the first ligands evaluated for TSPO expression in glioma patients [110,111,112].

The recently introduced TSPO ligand GE-180 labeled with [^18^F] offers an increased binding specificity and was tested in patients with gliomas [113] and neuroinflammatory diseases such as multiple sclerosis [114,115,116]. Regarding the delineation of glioma extent, it has been demonstrated that the [^18^F]GE-180 uptake volume is significantly larger than the volume of contrast enhancement [113,117]. However, when comparing [^18^F]FET with [^18^F]GE-180 uptake volumes intraindividually in terms of spatial distribution, the overlap is only moderate (Dice similarity coefficient, 0.55) despite comparable tumor volumes [117]. These differences might help to characterize glioma heterogeneity and warrant further studies with spatial correlation of imaging findings of [^18^F]FET uptake to [^18^F]GE-180 uptake with neuropathology.

### 3.4. PET Imaging of Cellular Proliferation

The radiolabeled nucleoside 3′-deoxy-3′-[^18^F]fluorothymidine ([^18^F]FLT) is a pyrimidine analogue. It is used to evaluate cellular proliferation because of its rapid incorporation into newly synthesized DNA [118]. [^18^F]FLT is trapped intracellularly after phosphorylation by the thymidine kinase-1, a cytoplasmatic enzyme expressed during cell proliferation [46,119]. However, the requirement of a disrupted blood-brain barrier for [^18^F]FLT uptake may limit its diagnostic use [2,120]. For example, in terms of tumor detection and delineation, [^18^F]FLT PET was less sensitive than [^11^C]MET PET to detect WHO grade II gliomas, which usually show no contrast enhancement [121]. Furthermore, a meta-analysis evaluating the value of [^18^F]FLT PET for the diagnosis of glioma recurrence based on approximately 800 patients showed no superiority of [^18^F]FLT (pooled sensitivity, 82%; pooled specificity, 76%) compared to [^18^F]FDG (pooled sensitivity, 78%; pooled specificity, 77%) [122].

On the other hand, [^18^F]FLT PET seems to be useful for the assessment of response to antiangiogenic therapy with bevacizumab in patients with recurrent malignant glioma. [^18^F]FLT PET was able to identify a reduction of proliferative activity in responding patients with favorable outcome as an indicator for response compared to metabolic non-responders [123,124,125]. Furthermore, [^18^F]FLT PET was used in patients with malignant melanoma brain metastases treated with targeted therapy or immunotherapy using checkpoint inhibitors [126]. In that study, responding patients showed a clearer reduction of proliferative activity as assessed by [^18^F]FLT PET than the decrease of contrast enhancement on standard MRI.

### 3.5. PET Imaging of Tumor Hypoxia

Hypoxia is a key factor in treatment outcome in various cancers, including glioma. It has been shown that hypoxia is associated with tumor persistence and resistance to cancer treatment [127]. To further evaluate this phenomenon using PET, the tracer [^18^F]fluoromisonidazole ([^18^F]FMISO) has been developed, which is trapped in hypoxic but viable cells [46,128,129]. It has been demonstrated in glioblastoma patients that [^18^F]FMISO PET delineates additional hypoxic tumor subregions which exceed the contrast-enhancing tumor parts, indicating that hypoxia may induce peripheral tumor growth [130]. A subsequent study showed that the metabolically active tumor volume in [^11^C]MET PET strongly correlated with the hypoxic volume defined by [^18^F]FMISO [131]. Importantly, the tumor area on [^11^C]MET PET exceeded the area of the contrast enhancement on MRI in the range of 20–30%.

More recently, [^18^F]FMISO PET has been used for monitoring the effects of antiangiogenic therapy with bevacizumab in patients with recurrent malignant glioma [132,133]. It was shown that patients who had a response in both contrast-enhanced MRI and [^18^F]FMISO PET had significantly longer survival than patients who responded on MRI only [133].

[^18^F]-labeled flouroazomycin arabinoside ([^18^F]FAZA) may be a promising alternative to [^18^F]FMISO offering an improved tumor-to-background ratio due to faster blood clearance [134,135]. Preliminary data in glioblastoma patients suggest that [^18^F]FAZA PET might be of value for radiotherapy response assessment [136].

### 3.6. PET Imaging of Tumor Perfusion

The evaluation of regional cerebral blood flow (rCBF) allows identifying brain tumors with high vascularization. rCBF can be measured by PET using [^15^O]-labeled water. However, [^15^O]H_2_O requires an on-site cyclotron because of its very short half-life (2 min) [137]. The development and easy accessibility of perfusion-weighted CT and MRI has led to various studies evaluating brain tumors outnumbering [^15^O]H_2_O PET studies by far. In direct comparisons, it has been shown that rCBF values differ considerably between CT- and MRI-based perfusion measurements and [^15^O]H_2_O PET [138,139].

In patients with malignant gliomas undergoing chemoradiation with nitrosoureas, a reduction of rCBF was evaluated using [^15^O]H_2_O PET, but data on subsequent survival as an indicator for treatment response are lacking [140,141].

### 3.7. PET Imaging of Angiogenesis

The vascular endothelial growth factor (VEGF) is overexpressed by most tumors, including brain tumors, and an important trigger for neovascularization [142]. Bevacizumab is a recombinant humanized monoclonal antibody against VEGF and appears to prolong progression-free survival and decrease steroid usage in patients with malignant glioma [142,143]. Based on these properties, [^89^Zr]-labeled bevacizumab PET imaging has been evaluated for tumoral drug accumulation in pediatric patients with diffuse intrinsic pontine glioma [144]. That study demonstrated intertumoral heterogeneity of drug accumulation and may aid in selecting those patients with the greatest chance of benefit from bevacizumab [144,145]. Other studies have used [^64^Cu]-labeled conjugates of VEGF with DOTA (1,4,7,10-tetra-azacylododecane *N*,*N*′,*N*′′,*N*′′′-tetraacetic acid) for PET imaging in animal models [146,147,148]. Similarly, these approaches suggest the clinical value for the identification of patients who will benefit from anti-VEGF therapy.

In adult glioblastoma patients, it has been demonstrated that accumulation of [^123^I]VEGF in the tumor region of glioblastomas can be assessed using single photon emission computed tomography (SPECT) imaging [149]. Importantly, high uptake of [^123^I]VEGF was able to identify glioblastoma patients with a poor clinical outcome.

## 4. Emerging Fields of PET Imaging

Currently, blockade of immune checkpoints and other immunotherapy options (i.e., vaccination strategies, oncolytic virus approaches, cell-based immunotherapy such as chimeric antigen receptor T-cells (CAR-T cells) are under evaluation in patients with brain cancer including glioma and brain metastases. Furthermore, recent study results suggest that newer-generation targeted therapies are a promising treatment option, especially in a subset of patients with brain metastases [150]. As a new treatment option, mutations of the isocitrate dehydrogenase (*IDH*) gene, which frequently occur in WHO grade II and III gliomas, have also gained interest as a potential treatment target [151,152]. All these promising treatment options impose new demands on brain imaging (e.g., imaging of immune reactions in the brain). The current body of literature suggests that PET has the potential to adapt to these needs. An overview is presented in Table 2.

### 4.1. PET Imaging of the Epidermal Growth Factor Receptor Family

Both the epidermal growth factor receptor (EGFR) and the human epidermal growth factor receptor 2 (HER2) are transmembrane protein receptors and belong to the EGFR family. These receptors are targets for various growth factors that mediate various cellular processes such as differentiation or proliferation. In clinical oncology, various gene mutations may lead to overexpression of these proteins and are associated with the development of a variety of cancers. Importantly, these mutations also play a significant role in various treatment options, including tyrosine kinase inhibitors and monoclonal antibodies targeting EGFR, HER2, or both [153], as well as for imaging. Especially the evaluation of a response to these targeted therapy options in patients with brain metastases (from melanoma, lung, or breast cancer) as well as in glioma patients is the indication with the highest clinical potential for EGFR- and HER2-targeted PET [154,155]. In recent years, radiolabeled EGFR and HER2 antibodies, as well as tyrosine kinase inhibitors, have been used as PET imaging agents.

**Figure 3:**
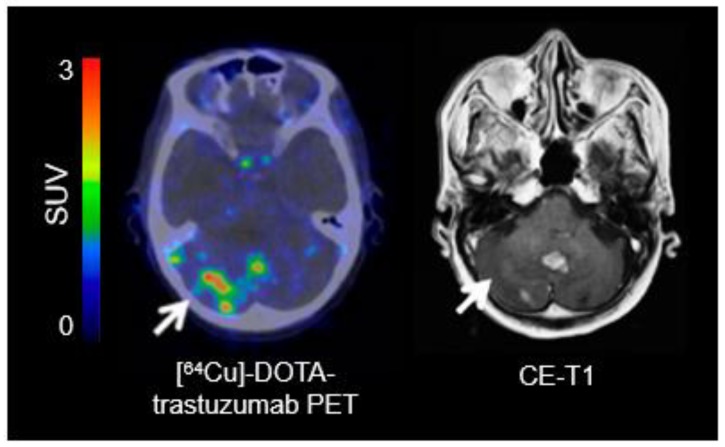
[^64^Cu]-DOTA-trastuzumab positron-emission tomography (PET) and contrast-enhanced magnetic resonance imaging (MRI) performed one day after initiation of treatment with trastuzumab in a patient with a human epidermal growth factor receptor 2 (HER2)-positive breast cancer with brain metastases. In single brain metastases, [^64^Cu]-DOTA-trastuzumab PET helps to improve lesion detection (arrow) (modified from Tamura et al. [160], with permission from the Society of Nuclear Medicine and Molecular Imaging).

For identifying EGFR overexpression, PET ligands such as [^11^C]erlotinib, [^11^C]PD153035, and [^89^Zr]Zr-DFO-nimotuzumab have been used [156,157,158]. The most relevant PET tracers for imaging of HER2 overexpression are [^64^Cu]DOTA-trastuzumab and [^89^Zr]pertuzumab (Figure 3) [159,160].

### 4.2. Immuno-Imaging: Immuno-PET and Imaging of T-Cells

In recent years, immunotherapy with antibodies directed against immune checkpoints such as the cytotoxic T-lymphocyte antigen-4 (CTLA-4; e.g., ipilimumab), the programmed cell death receptor-1 (PD-1; e.g., pembrolizumab, nivolumab), or the programmed cell death protein ligand 1 (PD-L1; e.g., atezolizumab) have gained paramount importance in clinical oncology and neuro-oncology. However, the efficacy and responsiveness of these agents may vary considerably among different cancer types and across individuals. Biomarkers obtained from tumor tissue, such as PD-1 and PD-L1 expression, can help to select patients. However, these tissue biomarkers are limited, and some patients show no response even if the target is present [161]. Therefore, the significance of PET for predicting response to immunotherapy and patient selection increases. Among other methods, immuno-PET combines antibodies or antibody fragments with a radionuclide and takes advantage of the specificity and affinity of antibodies and the sensitivity of PET [162]. Generally, targets for immuno-PET can be T-cell markers (e.g., CD4^+^, CD8^+^), immune checkpoints (e.g., CTLA-4, PD-1, PD-L1), or biomarkers of the immune response (e.g., interferon-γ, interleukin-2) [23].

First-in-human studies suggest that targeting PD-1 with [^89^Zr]nivolumab [163] or PD-L1 with [^89^Zr]atezolizumab [164] is useful as imaging biomarkers to non-invasively evaluate the expression of these immune checkpoints in patients with extra- and intracranial cancer [164,165]. Engineered target-binding proteins (adnectins) for PD-L1 ligands such as [^18^F]BMS-986192 are currently under evaluation [166].

**Figure 4:**
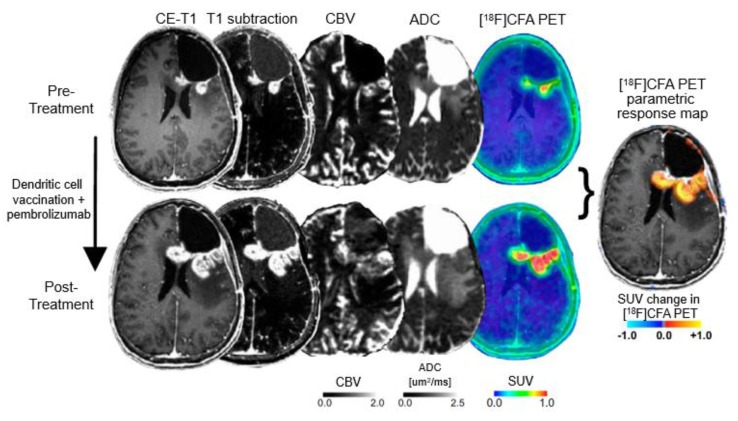
Detection of immune response in a patient with recurrent glioblastoma using 2-chloro-2′-deoxy-2′-[^18^F]fluoro-9-b-D-arabinofuranosyl-adenine ([^18^F]CFA) positron-emission tomography (PET) and advanced magnetic resonance imaging (MRI) before (upper panel) and after treatment with dendritic cell vaccination and programmed cell death receptor-1 (PD-1) blockade using pembrolizumab (lower panel). Following treatment, [^18^F]CFA uptake is considerably increased, indicating an immune-cell infiltration, and helps distinguishing tumor progression from inflammation (modified from Antonios et al. [169], with permission from the National Academy of Sciences).

Tumor-infiltrating T-cells (such as CD8^+^) play an essential role in the activation of immune cells in response to checkpoint inhibition [167]. Recently, it has been shown that a radiolabeled [^89^Zr]IAB22M2C has the potential to visualize CD8^+^ T-cell-enriched tumor tissue [168]. The assessment of immune cells infiltrating tumors has also been investigated with PET using radiolabeled clofarabine (2-chloro-2′-deoxy-2′-[^18^F]fluoro-9-b-D-arabinofuranosyl-adenine; [^18^F]CFA) [169]. [^18^F]CFA is a substrate for the enzyme deoxy-cytidine kinase, which is overexpressed in immune cells such as CD8^+^ T-cells [169]. Importantly, [^18^F]CFA PET has shown a great clinical potential to localize and quantify immune responses in glioblastoma patients undergoing dendritic cell vaccination treatment combined with immune checkpoint blockade (Figure 4) [169].

Another interesting approach is the transfection of immune cells with a reporter gene that encodes a protein that can be specifically targeted by a radiolabeled reporter probe [170]. Imaging of reporter gene expression of cells transfected with the herpes simplex virus type 1 thymidine kinase reporter gene has been demonstrated using 9-[4-[^18^F]fluoro-3-(hydroxymethyl)butyl]guanine ([^18^F]FHBG) [171]. Moreover, this technique has the potential to image recently introduced cell-based therapies with CAR-T cells [23]. Accordingly, a study with recurrent high-grade glioma patients suggested that [^18^F]FHBG PET can detect reporter gene expression in CAR-engineered cytotoxic T-lymphocytes [172].

### 4.3. PET Imaging of Isocitrate Dehydrogenase Mutations

In patients with malignant glioma, the modest outcome improvement following both standard therapy (i.e., chemoradiation with temozolomide) and newer treatment options (e.g., tumor-treating fields) has prompted various efforts to identify molecules that are fundamental to regulate tumor progression and provide additional options for personalized therapy in this group of patients.

Accordingly, the enzyme isocitrate dehydrogenase (IDH) has gained interest as a potential target. IDH is an enzyme of the Krebs cycle, catalyzing the oxidative decarboxylation of isocitrate to alpha-ketoglutarate. Mutations in the *IDH1* and *IDH2* gene, frequently occurring in WHO grade II or III astrocytomas and oligodendrogliomas, result in a significant increase of the oncometabolite 2-hydroxyglutarate (2-HG) [173,174]. In cells with IDH mutant enzymes, the accumulation of 2-HG alters several downstream cellular activities, causing epigenetic dysregulation and, consequently, a block in cellular differentiation, leading to oncogenesis [175].

Therefore, mutant IDH proteins are highly attractive targets for inhibitory drugs. In glioma patients, selective oral IDH inhibitors of IDH1 (i.e., ivosidenib, BAY-1436032), pan-IDH1/2 (i.e., AG-881), and vaccination strategies targeting the IDH1R132H mutation are currently under clinical evaluation. Initial results predominantly from phase-1 studies are promising and suggest that these inhibitors are safe and have antitumoral activity [151,152,176]. Although immunohistochemistry and genomic sequencing are the methods of choice for the detection of an IDH mutation, these techniques are invasive and are not appropriate for treatment monitoring, which requires continual assessment. Furthermore, the use of magnetic resonance spectroscopy (MRS) to non-invasively evaluate 2-HG is technically challenging, and may be false-positive in 20% of cases [177,178].

Newer PET probes for imaging mutant IDH expression in gliomas may be an alternative imaging method. Recent radiochemical developments suggest that triazinediamine or butyl-phenyl sulfonamide analogs labeled with [^18^F] are promising candidate radiotracers for noninvasive PET imaging of IDH mutations in gliomas [179,180]. Furthermore, a [^18^F]-labeled IDH1 inhibitor (AGI-5198) has also been investigated [181]. Interestingly, Koyaso and colleagues demonstrated that [^11^C]acetate uptake in IDH mutant cells is significantly higher than in IDH wild-type cells because of metabolic trapping [182]. Taken together, further efforts to translate these promising approaches for IDH imaging into clinical use are warranted.

### 4.4. PET-Based Theranostics

The combination of therapeutics and diagnostics, also termed as theranostics, supports the concept of precision oncology. One PET-based theranostic approach is the peptide receptor radionuclide therapy (PRRT), in which overexpressed tumor-specific receptors are used as a therapeutic target. By exchanging the radionuclide used for diagnostic PET such as [^68^Ga] with a radiation source, typically *ß*-emitters like [^177^Lu] or [^90^Y], the same PET tracer can be used for therapy. Although there are currently no theranostic approaches clinically established for gliomas or brain metastases, there are promising concepts. For example in patients with glioblastoma, a potential target is the overexpressed chemokine receptor-4 (CXCR4) which is associated with a poor clinical outcome [183,184,185]. Visualization of CXCR4 expression using diagnostic PET with CXCR4-directed [^68^Ga]Pentixafor^®^ has been demonstrated in glioblastoma patients [186].

Another potential target is the prostate-specific membrane antigen (PSMA) which may be overexpressed in prostate cancer and also in predominantly malignant gliomas. Diagnostic PET imaging of PSMA expression in patients with malignant glioma can be obtained using [^68^Ga]-labeled PSMA ligands [187,188,189]. The use of the theranostic agent [^177^Lu]-PSMA-617 has demonstrated favorable safety and efficacy in patients with advanced prostate cancer, indicating the potential to be also of value for patients with malignant glioma [190]. Nevertheless, the potential of theranostics needs to be further evaluated in patients with malignant gliomas.

## 5. Discussion

Anatomical MRI is currently the method of choice for neuroimaging of brain tumors, but PET complements this technique and provides important biological information that cannot be obtained from anatomical MRI alone. Currently, best-established PET tracers in neuro-oncology are radiolabeled amino acids targeting L-system transporters. However, a considerable number of other PET tracers have been developed for brain tumor patients and allow the evaluation of a wide range of biochemical processes. A variety of PET biomarkers offers the potential to play a clinically significant role for the monitoring of newer treatment options such as targeted therapy and immunotherapy, e.g., by providing an early assessment of response to these options, and for a more accurate differentiation of viable tumor from treatment-related changes. A major current shortcoming is the lack of large, prospective clinical trials in patients with both glioma and brain metastases for many of these PET tracers. Despite encouraging early study results in the field, it has to be further demonstrated that these tracers improve considerably patient management and outcome.

## Figures and Tables

**Table 1:** Frequently used PET tracers for the delineation of tumor extent, diagnosis of treatment-related changes, and the assessment of treatment response in brain tumor imaging.

Imaging Target and Corresponding Tracers	Delineation of Tumor Extent	Diagnosis of Treatment-Related Changes	Assessment of Treatment Response
**Glucose metabolism**			
[^18^F]FDG	-	+	-
**Amino acid transport**			
[^18^F]FET	++	++ ^1^	++ ^2^
[^11^C]MET	++	+	++
[^18^F]FDOPA	++	++	++
[^11^C]AMT	(++)	(++)	n.a.
[^18^F]FACBC	(++)	n.a.	n.a.
**Mitochondrial translocator protein (TSPO)**			
[^18^F]GE-180	unclear	n.a.	n.a.
**Cellular proliferation**			
[^18^F]FLT	-	+	++ ^3^
**Hypoxia**			
[^18^F]FMISO	n.a.	n.a.	(++) ^3^
[^18^F]FAZA	n.a.	n.a.	(++) ^3^
**Perfusion**			
[^15^O]H_2_O	n.a.	n.a.	n.a.
**Angiogenesis**			
[^89^Zr]bevacizumab	n.a.	n.a.	n.a.

++ = high diagnostic accuracy; (++) = high diagnostic accuracy, but limited data available; + = limited diagnostic accuracy; - = not helpful; ^1^ = increased accuracy when using dynamic [^18^F]FET PET; ^2^ = in enhancing and non-enhancing tumors; ^3^ = in patients undergoing antiangiogenic treatment with bevacizumab; [^11^C]AMT = α-[^11^C]-methyl-L-tryptophan; [^18^F]FACB = anti-1-amino-3-[^18^F]fluorocyclobutane-1-carboxylic acid; [^18^F]FAZA = [^18^F]flouroazomycin arabinoside; [^18^F]FDG = [^18^F]-2-fluoro-2-deoxy-D-glucose; [^18^F]FDOPA = 3,4-dihydroxy-6-[^18^F]fluoro-L-phenylalanine; [^18^F]FET = O-(2-[^18^F]fluoroethyl)-L-tyrosine; [^18^F]FLT = 3′-deoxy-3′-[^18^F]flurothymidine; [^18^F]FMISO = [^18^F]fluoromisonidazole; [^15^O]H_2_O = radiolabeled water; [^11^C]MET = [^11^C]methyl-L-methionine; n.a. = only preliminary or no data available.

**Table 2:** Promising PET tracers for the evaluation of newer treatment options.

Tracer	Target	Mechanism
**Imaging of the EGFR family**		
[^11^C]erlotinib	EGFR	TKI-mediated imaging
[^89^Zr]Zr-DFO-nimotuzumab	EGFR	Antibody-mediated imaging
[^11^C]PD153035	EGFR	TKI-mediated imaging
[^89^Zr]pertuzumab	HER2	Antibody-mediated imaging
[^64^Cu]-DOTA-trastuzumab	HER2	Antibody-mediated imaging
**Immuno-Imaging**		
[^89^Zr]nivolumab	PD-1	Antibody-mediated imaging
[^89^Zr]atezolizumab	PD-L1	Antibody-mediated imaging
[^18^F]BMS-986192	PD-L1	PET imaging using an engineered target-binding protein (adnectin)
[^89^Zr]IAB22M2C	CD8+ T-cells	Antibody fragment-mediated imaging
[^18^F]CFA	DCK	Targeting of the deoxy-cytidine kinase
[^18^F]FHBG	HSV1-tk	Imaging of reporter gene expression
**Imaging of IDH mutations**		
[^18^F]AGI-5198	IDH-mutant cells	Imaging of the mutant IDH enzyme using a radiolabeled IDH1 inhibitor
[^18^F]-labeled triazinediamine analogue	IDH-mutant cells	Imaging of the mutant IDH enzyme
Radiolabeled butyl-phenyl sulfonamide	IDH-mutant cells	Imaging of the mutant IDH enzyme
[^11^C]acetate	IDH-mutant cells	Metabolic trapping of the tracer in IDH-mutant cells

[^18^F]CFA = 2-chloro-2′-deoxy-2′-[^18^F]fluoro-9-b-D-arabinofuranosyl-adenine; DCK = deoxy-cytidine kinase EGFR = epidermal growth factor receptor; [^18^F]FHBG = 9-[4-[^18^F]fluoro-3-(hydroxymethyl)butyl]guanine; HER2 = human epidermal growth factor receptor 2; HSV1-tk = herpes simplex virus type 1 thymidine kinase; IDH = isocitrate dehydrogenase-1 or -2; PD-1 = programmed cell death receptor-1; PD-L1 = programmed cell death protein ligand 1; TKI = tyrosine kinase inhibitor.
